# Ruminal Fermentation of Anti-Methanogenic Nitrate- and Nitro-Containing Forages *In Vitro*

**DOI:** 10.3389/fvets.2016.00062

**Published:** 2016-08-11

**Authors:** Robin C. Anderson, Laura H. Ripley, Jan G. P. Bowman, Todd R. Callaway, Kenneth J. Genovese, Ross C. Beier, Roger B. Harvey, David J. Nisbet

**Affiliations:** ^1^Food and Feed Safety Research Unit, United States Department of Agriculture Agricultural Research Service, College Station, TX, USA; ^2^Department of Animal and Range Sciences, Montana State University, Bozeman, MT, USA

**Keywords:** alfalfa, anti-methanogenic, barley, nitrate, nitrocompound, milkvetch, rumen

## Abstract

Nitrate, 3-nitro-1-propionic acid (NPA) and 3-nitro-1-propanol (NPOH) can accumulate in forages and be poisonous to animals if consumed in high enough amounts. These chemicals are also recognized as potent anti-methanogenic compounds, but plants naturally containing these chemicals have been studied little in this regard. Presently, we found that nitrate-, NPA-, or NPOH-containing forages effectively decreased methane production, by 35–87%, during *in vitro* fermentation by mixed cultures of ruminal microbes compared to fermentation by cultures incubated similarly with alfalfa. Methane production was further decreased during the incubation of mixed cultures also inoculated with *Denitrobacterium detoxificans*, a ruminal bacterium known to metabolize nitrate, NPA, and NPOH. Inhibition of methanogens within the mixed cultures was greatest with the NPA- and NPOH-containing forages. Hydrogen accumulated in all the mixed cultures incubated with forages containing nitrate, NPA or NPOH and was dramatically higher, exceeding 40 μmol hydrogen/mL, in mixed cultures incubated with NPA-containing forage but not inoculated with *D. detoxificans*. This possibly reflects the inhibition of hydrogenase-catalyzed uptake of hydrogen produced *via* conversion of 50 μmol added formate per milliliter to hydrogen. Accumulations of volatile fatty acids revealed compensatory changes in fermentation in mixed cultures incubated with the nitrate-, NPA-, and NPOH-containing forages as evidenced by lower accumulations of acetate, and in some cases, higher accumulations of butyrate and lower accumulations of ammonia, iso-buytrate, and iso-valerate compared to cultures incubated with alfalfa. Results reveal that nitrate, NPA, and NPOH that accumulate naturally in forages can be made available within ruminal incubations to inhibit methanogenesis. Further research is warranted to determine if diets can be formulated with nitrate-, NPA-, and NPOH-containing forages to achieve efficacious mitigation in ruminant methane emissions without adversely affecting fermentative efficiency or risking toxicity to animals.

## Introduction

Nitrate and the naturally occurring nitrocompounds, 3-nitro-1-propionic acid (NPA) and 3-nitro-1-propanol (NPOH), are oxidized nitrogen compounds that can accumulate to toxic levels in certain forages and feedstuffs. Toxicologically, nitrate exerts its effects by first being biologically reduced to the toxic intermediate nitrite by bacteria within the rumen, and upon subsequent absorption, the nitrite complexes with the host’s hemoglobin to form methemoglobin, which thus loses its oxygen carrying capacity. Consequently, severely poisoned animals suffer asphyxiation (1). Poisoning by NPA and NPOH occurs differently, *via* inactivation of cellular succinate dehydrogenase activity, which thus inhibits cellular respiration (2). Nitrate, NPA, and NPOH are also recognized as potent inhibitors of ruminal methane production, a microbiological process that can result in the loss of 4–12% of the gross energy consumed by concentrate- or forage-fed cattle, respectively (3). Production of methane from ruminant sources also contributes nearly 20% of the total U.S. emissions of methane, which is a potent greenhouse gas (4), and strategies are sought to reduce the economic and environmental impact of this digestive inefficiency.

A number of recent reviews have been published on the methane-inhibiting potential and the toxicity of nitrate, its reduced intermediate, nitrite (1, 5, 6), as have reviews on similar aspects of NPA, NPOH, and a number of other nitroalkanes (1, 2, 6, 7). Most of the research findings discussed in these reviews have been based on studies investigating effects of the specific chemicals themselves on the rumen ecosystem and the host with fewer studies investigating the effects of plants containing the compounds.

In the case of nitrate, for instance, the use of nitrate salts as feed supplements to reduce methane emissions from ruminants has been investigated in a number of studies, as recently reviewed (1, 5, 6). Mechanistically, nitrate is attractive as a methane-inhibitor because of its conversion to nitrite, which is further converted to ammonia by a process that consumes reducing equivalents that otherwise would be used to reduce carbon dioxide to produce methane. However, concerns persist that the potential accumulation of the toxic intermediate nitrite, which if occurring too rapidly and at too high a concentration within the rumen, may inhibit microbes that are important for fiber digestion, and if absorption is sufficient to cause methemoglobinemia can risk poisoning of the host (8). This may be problematic when using sodium or potassium salts as these are very rapidly converted to nitrite, which may accumulate to toxic levels before it can be further metabolized to ammonia. Potential approaches to lessen the rate of nitrite accumulation within the rumen are to use more resistant salts, such as calcium salts, or to use encapsulated sources (9), but these may add cost to their commercial application.

With respect to the effects of nitrocompounds on rumen methane production, only a few studies have examined the natural compounds, NPA and NPOH (10, 11), with most reporting results from studies examining the xenobiotic nitroalkanes (12–22) and nitroxy compounds (23–30). Mechanistically, the natural and synthetic nitroalkanes have been suggested to inhibit ruminal metabolism of hydrogen and formate, substrates used for rumen methanogenesis, although the mechanisms have not been defined (11). The nitrooxy compounds are reported to inhibit methyl-coenzyme M reductase of methanogenic bacteria (24, 25).

From a practical standpoint, the xenobiotic nitrocompounds will likely require extensive testing to address toxicity and safety concerns. Moreover, the known or presumed microbial metabolic by-products of nitroalkanes, such as nitroethane, 2-nitroethanol, and 2-nitro-1-propanol (aminoethane, ethanolamine, and aminopropanol), are anticipated to be of little nutritional value for the ruminant host. We are not aware of reports on the fate of the nitrooxy nitrocompounds. On the other hand, NPA is known to be metabolized by ruminal microbes to β-alanine (31), a non-essential amino acid that may be metabolized in the rumen to sources of carbon, nitrogen, and energy (32). Thus, it is likely that when occurring in their natural state as secondary plant compounds, forages containing NPA or NPOH may be viewed more favorably by regulatory agencies. While the toxicity of NPOH may limit its application as a methane-inhibitor, NPA is not as toxic and has been safely fed to ruminants in various feedstuffs, most notably as cured Crownvetch (*Coronilla varia*) hay (33), indicating that its toxicity may be managed by controlled feeding. Other leguminous forages can accumulate NPA, and these include *Indigofera* and a number of different species and varieties of *Astragalus*, commonly named milkvetches, some which may also accumulate NPOH (2), but little is known about how these forages may affect ruminal fermentation. The objectives of the present studies were to assess the methane-inhibiting activity of forages available to us containing nitrate, NPA, or NPOH and to examine their effects of ruminal fermentation and methanogen numbers *in vitro*.

## Materials and Methods

### Forage Sources

Alfalfa (*Medicago sativa*) used as a control was grown and harvested by a farmer in College Station, TX, USA and was purchased locally. The low and high nitrate-containing barley (*Hordeum vulgare*) containing 0.23 and 1.69% nitrate, respectively, were provided by Dr. Jan G. P. Bowman and have been studied for their potential genetic effects on forage quality (34). *Astragalus canadensis* containing predominantly the tri- and di-NPA glucopyranose esters karakin (1,2,6-tri-*O*-[3-nitropropanyl]-β-d-glucopyranose) and cibarian (1,6-di-*O*-[3-nitropropanyl]-β-d-glucopyranose) at approximately 1.6% of plant dry matter and *Astragalus miser* containing ether glycosides of NPOH, predominantly as miserotoxin (3-nitro-1-propyl-β-d-glucopyranoside) at approximately 2–5% plant dry matter, were graciously provided by Dr. Walter Majak (Agriculture and Agrifood Canada, Kamploops, BC, Canada). Procedures for the collection and measurement of conjugated nitrocompounds in the forages have been described (35, 36). For the present study, chemical composition and nitrate concentration for each of the forages was determined by the Soil, Water and Forage Testing Laboratory at the Texas AgriLife Extension Services’ Department of Soil and Crop Sciences (37), and these data are presented in Table 1.

**Table 1:** **Composition of alfalfa, high and low nitrate-containing barley, and NPA- and NPOH-containing milkvetches**.

	Alfalfa	High nitrate-containing barley	Low nitrate-containing barley	NPA-containing milkvetch	NPOH-containing milkvetch
Crude protein (%)	17.3	13.8	6.4	10.0	9.6
Digestible crude protein (%)	12.6	9.4	2.5	5.8	5.4
Acid detergent fiber (%)	35.5	35.4	31.4	32.4	34.8
Neutral detergent fiber (%)	38.8	45.0	46.9	38.3	37.8
Total digestible nutrients (%)	60.2	59.1	59.5	60.0	58.1
Net energy lactation (Mcal/kg)	1.36	1.32	1.34	1.34	1.30
Net energy maintenance (Mcal/kg)	1.45	1.43	1.43	1.45	1.39
Net energy gain (Mcal/kg)	0.73	0.70	0.70	0.73	0.68
*In vitro* true digestibility (%)	75.7	80.4	68.5	85.3	82.3
Ash (%)	10.2	9.3	5.4	7.4	7.9
Relative feed value	146.7	126.8	127.7	154.7	152.0
Nitrate^a^ (%)	0.16	1.69	0.23	0.15	0.03
Mineral analysis (NIR)					
Calcium	1.22	0.69	0.48	0.72	0.77
Magnesium	0.29	0.15	0.04	0.11	0.11
Phosphorus	0.39	0.29	0.16	0.25	0.23
Potassium	2.92	2.23	1.52	1.64	1.50

*^a^Amounts of nitrate potentially available in each tube for mixed cultures of ruminal microbes incubated without being inoculated with *D. detoxificans* (experiment 1) are 1.3, 13.6, 1.8, 1.2, and 0.2 μmol/mL of incubation fluid for alfalfa, high nitrate-containing barley, low nitrate-containing barley, NPA-containing milkvetch, and NPOH-containing milkvetch, respectively. Amounts of nitrate potentially available in each tube for mixed cultures of ruminal microbes in experiment 2 are estimated to be 16.7% less to account for additional 2 mL volume with *D. detoxificans* inoculation*.

### Mixed Culture of Ruminal Microbes

Two separate *in vitro* rumen incubation experiments were conducted using freshly collected ruminal fluid obtained at 1000 hours (2 h after morning feeding) from a rumen-cannulated Holstein cow (approximately 660 kg) maintained on 50:50 corn-based concentrate:alfalfa diet, supplemented with a commercially available mineral mix (Producers CO-OP, Bryan, TX, USA). All procedures with the cow were conducted in accordance with the Southern Plains Agricultural Research Center’s approved Animal Care and Use protocol. The ruminal fluid was strained through a nylon paint strainer during collection into insulated containers until completely full, then capped and returned to the laboratory within 30 min of collection for immediate use. At the laboratory, the ruminal fluid was amended to achieve 50 mM sodium formate and then distributed (within 30 min of collection) in 10 mL volumes to two sets of 18 × 150-mm crimp-top culture tubes preloaded in triplicate with 0.5 g (92–96% dry matter) of each test forage previously ground to pass a 4 mm Willey Mill screen. The ruminal fluid was kept under a 100% carbon dioxide atmosphere during preparation and transfer at the laboratory to maintain anaerobiosis, and tubes were immediately closed with rubber stoppers and crimped to prevent leakage during subsequent 24-h incubation at 39°C in upright position without agitation. In experiment 1, no further additions were made. In experiment 2, which was conducted concurrently with experiment 1, the loaded and capped tubes were inoculated with approximately 2 × 100 cells of a 72-h old culture of the NPA-, NPOH-, and nitrate-metabolizing ruminal bacterium *Denitrobacterium detoxificans* strain NPOH1, grown previously in 50-mL nitro-supplemented Medium B as described by Anderson and Rasmussen (10). Inoculations were accomplished *via* injection of 2 mL culture volume into each tube through the rubber stopper using a needle just prior to the start of incubation. At the end of the incubation period, 1 mL of atmosphere from the headspace of each tube was collected *via* a 1-mL glass syringe and injected into a Gow-Mac series 580 gas chromatograph (Gow-Mac Instrument, Bridgewater, NJ, USA) equipped with a HaySep Q column heated to 60°C and operated with Argon as the carrier gas flowing at 25 mL/min. Methane and hydrogen were measured with a thermal conductivity detector. Gas volumes were measured *via* volume displacement using a 30-cc lubricated glass syringe. Molar concentrations of hydrogen and methane were calculated using the Idea Gas Laws and are expressed as micromole per milliliter of incubation fluid. Fluid samples collected at the end of incubation were used for colorimetric measurement of ammonia, nitrate, nitrite, NPA, and NPOH (38–41) and for gas chromatographic measurement of volatile fatty acids (42). Most probable numbers (MPN) of methanogens, expressed as log_10_ cells/mL incubation fluid, at the end of the incubations were determined as described by Saengkerdsub et al. (43) except using an Association of Official Analytical Chemists’ 3-tube MPN table (44).

### Statistical Analysis

Statistical comparisons between mixed cultures incubated with the different forages were made within experiment to avoid confounding effects of volume differences between the two experiments. Because each resultant population had the opportunity to respond independently, each was considered an independent experimental unit. Tests for effects of forage type on accumulations of hydrogen, methane, ammonia, and volatile fatty acids after 24-h incubation of mixed cultures were conducted using a completely randomized analysis of variance (*n* = 3/forage type) with an LSD separation of means (Statistix 10 Analytical Software, Tallahassee, FL, USA). MPN estimates were similarly analyzed for effects of forage type within experiments 1 and 2 using a completely randomized analysis of variance with an LSD separation of means except using measurements made from only two of the three replicate tubes per each forage type (*n* = 2/forage type) incubated with mixed cultures of ruminal microbes.

## Results

### Experiment 1 (Incubation of Mixed Cultures without *D. detoxificans* Inoculation)

Total volume of gas produced tended to differ between mixed cultures of ruminal microbes incubated with the different forages (Table 2). Methane accumulations were 79, 85, and 35% lower after 24 h in mixed cultures incubated with the high nitrate-containing barley and the NPA- and NPOH-containing milkvetches, respectively, when compared to accumulations in mixed cultures incubated with alfalfa (Table 2). Hydrogen accumulations were dramatically higher in mixed cultures incubated with the NPA-containing milkvetch and were lowest in mixed cultures incubated with alfalfa or the low nitrate-containing barley, with accumulations in mixed cultures incubated with the high nitrate-containing barley and NPOH-containing milkvetch being intermediate (Table 2). Differences in accumulations of all volatile fatty acids except valerate were observed, and these data are presented in Table 2. Differences in ratios of acetate to propionate were also observed, being highest with mixed cultures incubated with alfalfa and 10–31% lower in mixed cultures incubated with the other forages (Table 2). Ammonia accumulations differed between the mixed cultures incubated with concentrations being highest in the mixed cultures incubated with high nitrate-containing barley and lowest in mixed cultures incubated with NPA-containing milkvetch. Residual concentrations of nitrate, nitrite, and the nitrocompounds at the end of the 24-h incubations were not tested for differences between the different forages because initial concentrations were not the same. However, measurements revealed that residual concentrations of nitrate and nitrite in fluids from all incubations were below 1.3 and 0.04 μmol/mL, respectively. Residual concentrations of NPA and NPOH were 4.4 and 8.7 μmol/mL, respectively. MPN of methanogens were highest in mixed cultures incubated with alfalfa, lowest in mixed cultures incubated with the NPA-containing milkvetch, and intermediate in mixed cultures incubated with NPOH-containing milkvetch and the high and low nitrate-containing barley.

**Table 2:** **Fermentation characteristics of alfalfa and select nitrate- and nitro-containing forages during incubation with mixed cultures of ruminal microbes in experiment 1 with 50 mM added sodium formate, but without inoculation with *Denitrobacterium detoxificans***.

	Alfalfa	High nitrate-containing barley	Low nitrate-containing barley	NPA-containing milkvetch	NPOH-containing milkvetch	*P* value	SEM
**Headspace measurements**							
Total gas, mL	46.0^b^	41.3^b^	54.0^a^	46.3^a,b^	46.7^a,b^	0.0529	2.463
Hydrogen, μmol/mL (kPa)	0.66^c^ (1.0)	3.06^b,c^ (4.7)	0.88^c^ (1.4)	40.15^a^ (61.6)	5.50^b^ (8.5)	<0.0001	0.956
Methane, μmol/mL	62.36^a^	13.12^c^	76.35^a^	9.52^c^	40.72^b^	<0.0001	4.680
**Fluid measurements**							
Acetate, μmol/mL	178.68^a^	138.06^c^	151.60^b^	125.61^d^	129.63^d^	<0.0001	2.617
Propionate, μmol/mL	63.74^a^	63.81^a^	60.35^b^	50.06^c^	66.96^a^	<0.0001	1.030
Butyrate, μmol/mL	38.66^c^	45.18^b^	55.42^a^	52.48^a^	53.97^a^	<0.0001	1.372
Iso-butyrate, μmol/mL	6.38^b^	7.81^a^	5.84^c^	4.83^d^	6.04^c^	<0.0001	0.089
Iso-valerate, μmol/mL	5.71^b^	6.44^a^	5.11^c^	4.04^d^	5.70^b^	<0.0001	0.066
Valerate, μmol/mL	8.45	11.95	8.65	8.82	9.5	0.4441	1.428
Total VFA, μmol/mL	301.63^a^	273.24^b^	286.96^a,b^	245.83^c^	271.86^b^	0.0003	5.211
Ratio of acetate to propionate	2.80^a^	2.16^c^	2.51^b^	2.51^b^	1.94^d^	<0.0001	0.030
Ammonia, μmol/mL	58.08^a,b^	63.25^a^	42.26^c,d^	38.54^d^	49.96^b,c^	0.0003	2.628
Numbers of methanogens, log_10_ cells/mL	3.01^a^	2.17^b,c^	2.36^a,b^	1.45^c^	2.80^a,b^	0.0196	0.209

*^a,b,c,d^Means within rows with unlike superscripts differ at P < 0.05*.

### Experiment 2 (Incubation of Mixed Cultures with *D. detoxificans* Inoculation)

Total gas volumes after 24-h incubation of mixed cultures of ruminal microbes that had been inoculated with *D. detoxificans* differed, with amounts produced being higher in mixed cultures incubated with alfalfa than in the mixed cultures incubated with the other forages (Table 3). Accumulations of methane and hydrogen also differed, with more methane accumulating in mixed cultures incubated with alfalfa than with the other forages, and with more hydrogen accumulating in mixed cultures incubated with the NPA-containing milkvetch than with the other forages (Table 3). Differences in accumulations of all volatile fatty acids except propionate were observed, and these data are presented in Table 3. Differences in total volatile fatty acid accumulations were observed, with lower accumulations observed for mixed cultures incubated with the NPA- and NPOH-containing milkvetches than in the other forages, mainly due to lower accumulations of acetate (Table 3). Lower accumulations of acetate observed in mixed cultures incubated with the NPA-, NPOH-, and nitrate-containing forages, than in mixed cultures incubated with alfalfa, are also reflected in the ratios of acetate to propionate (Table 3). Accumulations of ammonia were highest in mixed cultures incubated with alfalfa, lowest in mixed cultures incubated with the NPA- and NPOH-containing milkvetches, and intermediate in mixed cultures incubated with the high and low nitrate-containing barley (Table 3). Residual concentrations of nitrate and nitrite in fluids from all incubations were below 0.82 and 0.02 μmol/mL, respectively. Residual concentrations of NPA and NPOH were 3.6 and 7.2 μmol/mL, respectively. MPN of methanogens were higher in mixed cultures incubated with alfalfa and the high nitrate-containing barley than in mixed cultures incubated with the NPA- and NPOH-containing milkvetches and the low nitrate-containing barley (Table 3).

**Table 3:** **Fermentation characteristics of alfalfa and select nitrate- and nitro-containing forages during incubation with mixed cultures of ruminal microbes in experiment 2 with 50 mM added sodium formate and inoculation with *Denitrobacterium detoxificans***.

	Alfalfa	High nitrate-containing barley	Low nitrate-containing barley	NPA-containing milkvetch	NPOH-containing milkvetch	*P* value	SEM
**Headspace measurements**							
Total gas, mL	48.7^a^	30.3^b^	27.3^b^	19.3^b^	27.3^b^	0.0062	4.145
Hydrogen, μmol/mL (kPa)	1.07^b^ (1.7)	0.95^b^ (1.5)	0.92^b^ (1.4)	6.58^a^ (10.1)	4.18^a,b^ (6.4)	0.0392	1.308
Methane, μmol/mL	54.90^a^	2.35^b^	6.48^b^	0.80^b^	4.57^b^	<0.0001	1.951
**Fluid measurements**							
Acetate, μmol/mL	145.19^a^	123.08^b^	130.55^b^	97.99^c^	106.77^c^	<0.0001	2.967
Propionate, μmol/mL	54.54	57.30	55.35	53.54	59.70	0.3856	2.270
Butyrate, μmol/mL	34.34^b^	37.73^b^	48.07^a^	46.76^a^	48.98^a^	0.0093	2.698
Iso-butyrate, μmol/mL	6.07^b^	7.14^a^	5.97^b^	4.86^d^	5.40^c^	<0.0001	0.119
Iso-valerate, μmol/mL	5.37^b^	5.92^a^	5.41^b^	4.56^c^	5.28^b^	0.0013	0.150
Valerate, μmol/mL	7.69^b^	7.68^b^	8.30^a,b^	9.33^a^	9.14^a^	0.0138	0.335
Total VFA, μmol/mL	253.19^a^	238.85^a,b^	253.66^a^	217.04^c^	235.26^b^	0.0037	5.333
Ratio of acetate to propionate	2.66^a^	2.15^b,c^	2.40^a,b^	1.83^c^	1.79^c^	0.0020	0.121
Ammonia, μmol/mL	51.60^b^	60.87^a^	42.02^c^	34.73^d^	42.06^c^	<0.0001	1.479
Numbers of methanogens, log_10_ cells/mL	2.36^a^	2.38^a^	1.80^c^	1.27^c^	1.36^c^	0.0006	0.086

*^a,b,c,d^Means within rows with unlike superscripts differ at P < 0.05*.

## Discussion

As reviewed recently by Latham et al. (1), nitrate and certain short-chain nitrocompounds are known to be potent methane-inhibiting compounds, although their use in research studies has almost exclusively been done with commercially available chemical sources using various nitrate salts or chemically synthesized nitrocompounds. Results from the present study confirm that rumen methanogensis can be lowered with forages containing nitrate, NPA, and NPOH, thus indicating that these compounds were readily solubilized or otherwise made available within the incubation fluids to inhibit methanogenesis. Forage quality and digestibility can also affect ruminal methanogenesis (3), and thus quality differences between the forages in the present study may have contributed to differences in amounts of methane produced. However, the neutral detergent fiber, acid detergent fiber content, and the *in vitro* true digestibility of the nitrate- and nitro-containing forages differed no more than 10% and in some cases were higher than that of the alfalfa (Table 1). Accordingly, it is seems likely the differences in methane production were due largely to nitrate and the nitrocompounds contained in the barley and milkvetch forages.

An attractive aspect of using nitrate as a methane-inhibitor is that it can act as an energetically favorable alternative electron acceptor that consumes electrons at the expense of methanogenesis, thereby preserving energetic efficiencies of interspecies-hydrogen transfer thought to be beneficial for rumen digestive processes (1). In such cases, nitrate is reduced to nitrite, which in the rumen is predominantly reduced further to ammonia (45), with the process consuming eight electrons, the equivalent of 4 μmol of hydrogen, for each micromole of nitrate reduced to ammonia (1). The methane-inhibiting potential of nitrate, however, is largely dependent on sufficiently active nitrate-reducing ruminal microbes, such as those having been adapted to nitrate *via* prior exposure. For instance, Božic et al. (16) reported that methane production was not inhibited during an initial 24-h incubation of ruminal microbes with 16 μmol nitrate/mL but was inhibited upon subsequent transfer of this 24-h old population to a fresh nitrate-containing medium. Based on the amounts of nitrate in the forages used in the present study, initial concentrations of nitrate potentially available in the incubations in experiment 1 were estimated to be 1.3, 13.6, 1.8, 1.2, and 0.2 μmol/mL of incubation fluid for alfalfa, the high and low nitrate-containing barley, and the NPA- and NPOH-containing milkvetch, respectively. Potentially available nitrate concentrations for incubations in experiment 2 would be expected to be 16.7% less due to dilution of the incubation fluid that occurred with inoculation of *D. detoxificans*. Accordingly, except for the mixed cultures incubated with the high nitrate-containing barley, nitrate concentrations in incubations with the other forages would most likely have been too low to affect appreciable decreases in methane production. While nitrate *per se* is not particularly toxic to rumen methanogenic bacteria, the reduced intermediate, nitrite, is a potent inhibitor of methanogenesis, causing 50% decrease in methane-producing activity with concentrations as low as 0.5 μmol/mL (46). Thus, it is possible that nitrite may accumulate to concentrations directly inhibitory to methanogens when rates of nitrate reduction to nitrite exceed rates of nitrite reduction to ammonia. In experiment 1, the mixed cultures had no known prior exposure to nitrate, and therefore, rates of nitrate reduction to nitrite would be expected to proceed slowly at first but increase rapidly as a consequence of induction of nitrate-reducing activity and selection of nitrate-reducing microbes. Eventually, rates of nitrate reduction could far exceed the rates of nitrite reduction as the mixed cultures adapted, thus potentially allowing nitrite to accumulate to inhibitory concentrations in the mixed cultures in experiment 1, particularly for the mixed cultures incubated with the high nitrate-containing barley where higher nitrite accumulations could have persisted for a longer duration than in mixed cultures incubated with the other forages. However, nitrite concentrations, in all of the mixed cultures in both experiments 1 and 2, were below 0.04 μmol/mL in fluid samples measured at the end of the 24-h incubation period, which suggest that inhibitory concentrations of nitrite would have been temporary.

For mixed cultures in experiment 2 that had been inoculated with the competent nitrate-metabolizing bacterium *D. detoxificans*, rapid rates of nitrate and nitrite metabolism would be expected to have commenced sooner, and thus accumulations of nitrite would be expected to be lower and to persist for a shorter duration than in cultures of experiment 1. The lower accumulation of methane observed with mixed cultures incubated with the low nitrate-containing barley in experiment 2 is not readily explained, as the available nitrate (and subsequently nitrite) would be expected to be too low to affect appreciable inhibition in methanogenesis. It is possible that there may have been some carry over of residual nitrocompound with the 2-mL inoculum, but this seems unlikely as a potential carry over effect would have manifested itself in all the mixed cultures. Differences in MPN of methanogens in the *D. detoxificans*-inoculated cultures in experiment 2 were observed, being lower in mixed cultures incubated with the low nitrate-containing barley cultures, as well as in those incubated with the milkvetches, than in cultures incubated with the high nitrate-containing barley. In the latter case, the lower methane production is consistent with competitive consumption of electrons rather than direct inhibition of methanogens. The high and low nitrate-containing barley forages were different genotypes sampled at different stages of maturity, with the high nitrate-containing forage sampled at flowering (plant anthesis) and the low nitrate-containing forage sampled at peak forage yield (34). It is possible that maturation of the low nitrate-containing barley may have caused accumulations of reactive nitrogen derivatives or accumulations of oxidized sulfur-containing compounds, such as oxidized cysteine residues in Rubisco (47), which could potentially be metabolized to yield suitable electron acceptors for *D. detoxificans*. *D. detoxificans* is known to be able to respire anaerobically, oxidizing hydrogen, formate, or lactate to reduce nitrate, NPA, NPOH, as well as various other oxidized compounds, such as trimethylamine oxide and dimethyl sulfoxide (48, 49), but its ability to use other naturally occurring electron acceptors has not been thoroughly investigated.

In the case of the nitro-containing milkvetches, greater reduction in methane production was achieved in mixed cultures incubated with the NPA-containing milkvetch than with the NPOH-containing milkvetch. This likely reflects a more potent methane-inhibiting potential of NPA compared to NPOH, considering that nearly twice as much nitrocompound was potentially available in the incubations supplemented with the NPOH-containing milkvetch (8.7 and 7.2 μmol/mL in experiments 1 and 2, respectively) than in the NPA-containing milkvetch (4.4 and 3.6 μmol/mL, in experiments 1 and 2, respectively). Earlier work also indicated that NPA inhibited methane production more effectively than 2-nitro-1-propanol, a structural isomer of NPOH (11). In support of these observations, MPN estimates of methanogens revealed a differential response within the mixed cultures incubated with the NPA- or NPOH-containing milkvetch in experiment 1. In this case, mixed cultures incubated with the NPA-containing milkvetch had lower methanogen numbers than mixed cultures incubated with alfalfa as well as those incubated with NPOH-containing milkvetch and low nitrate-containing barley. Conversely, methanogen numbers in experiment 2 were found to be equivalently lower in mixed cultures incubated with the NPA- and NPOH-containing milkvetches when compared to mixed cultures incubated with alfalfa. It is recognized that when *D. detoxificans* is present, NPA and NPOH may transition, at least partially, from being direct inhibitors of methanogenesis to being used as alternative electron acceptors to support the growth of *D. detoxificans* (1).

For the mixed cultures incubated with the NPOH-containing milkvetch, and to a lesser extent with the NPA-containing milkvetch, there is evidence that inoculation with *D. detoxificans* may have promoted consumption of electrons, but this would have had little impact in limiting availability of electrons for methanogenesis. For instance, residual concentrations of NPA and NPOH at the end of the 24-h incubations with the NPA- and NPOH-containing milkvetches were 86 and 87% of initial concentrations in experiment 1 indicating metabolism of about 0.5–1.1 μmol NPA or NPOH/mL, respectively. Conversely, residual NPA and NPOH were 61 and 64% of initial concentrations in the incubations with NPA- and NPOH-containing milkvetches in experiment 2, which corresponds to metabolism of about 1.4 and 2.6 μmol NPA or NPOH/mL, respectively. Assuming that each micromole of NPA or NPOH reduced consumes six electrons or the equivalent of 3 μmol of hydrogen, based on stoichiometric estimates for the reduction of nitroethane by a *Clostridium pasteurianum* hydrogenase/ferredoxin system (50), the NPA and NPOH metabolized in this study would have consumed at most only 4.2 and 7.8 μmol hydrogen equivalents/mL, respectively. The lesser amounts of NPA and NPOH metabolized within the mixed cultures incubated with NPA- and NPOH-containing milkvetch incubations in experiment 1, which were not inoculated with *D. detoxificans* is not surprising, considering that *D. detoxificans* is usually present at low concentrations (<10^4^ cells/mL) in rumen populations having no prior nitrocompound exposure (51). Based on these considerations, the more potent methane-inhibiting effect observed in the mixed cultures inoculated with *D. detoxificans* and incubated with the NPOH-containing milkvetch cannot be explained solely by competitive consumption of reducing substrates for the reduction of NPOH. Thus, other modes of action must be operative, and this possibility warrants further investigation. It is also possible that populations of rumen microbes sufficiently adapted to higher concentrations of NPA or NPOH may be able to consume greater concentrations, reducing substrates to quantitatively impact methanogens. However, the toxicity of higher concentrations of NPA or NPOH may limit amounts of these nitrocompounds that can be fed in practical animal feeding situations.

In the present study, 50 μmol formate/mL was added to the incubations of both experiments to provide non-limiting amounts of reducing substrate to support the reduction of nitrate, NPA, or NPOH, which would be expected to yield ammonia, β-alanine, or 3-amino-1-propanol, respectively (1, 48–52). It was expected that most, if not all, of the added formate would be converted to hydrogen, which would subsequently serve as reducing substrate, as formate is usually converted to hydrogen *via* activity of microbial formate hydrogenlyase and formate dehydrogenase (53, 54). However, it is possible that some of the formate may have served as a reducing substrate itself as formate is a good substrate for methane production and for the reduction of nitrate and the nitrocompounds. In experiment 1, hydrogen accumulations in the mixed cultures incubated with the NPA- and NPOH-containing milkvetches were higher than in the mixed cultures incubated with alfalfa, thus indicating an effect of the nitrocompounds on hydrogen utilization. In the case of the mixed cultures incubated with the NPA-containing milkvetch, the higher accumulation of hydrogen, exceeding 40 μmol/mL incubation fluid, supports our expectation that considerable amounts of the 50-μmol/mL added formate was biotransformed into hydrogen. The high accumulation of hydrogen in the mixed cultures incubated with the NPA-containing milkvetch also suggests subsequent inhibition of hydrogen oxidation. In an earlier study, ruminal populations treated with NPA and other short-chain nitrocompounds (2-nitro-1-propanol, 2-nitroethanol, and nitroethane) and incubated without added *D. detoxificans* were found to inhibit the oxidation of hydrogen and formate, but mechanistic aspects of this inhibition have yet to be resolved (11). Formate concentrations were not measured in the present experiments, and thus the possibility that some residual formate may have been retained in these incubations cannot be excluded.

Differences in accumulations of volatile fatty acids were observed in the mixed cultures incubated with the different forages, thus reflecting differences in digestibility of the different forages due in part to the inhibition of methanogenesis and its role in maintaining low hydrogen concentrations. In both experiments 1 and 2, accumulations of total volatile fatty acids were nearly 30% lower in mixed cultures incubated with both NPA- and NPOH-containing milkvetches than in cultures incubated with alfalfa. Earlier work had reported modest inhibitory effects of 21 μmol/mL NPA and NPOH on total culturable rumen anaerobes, with decreases in viable cell counts being 32% or less from untreated counts (1.8 × 10^9^ colony-forming U/mL), although the specific microbes inhibited were not characterized (31). More severe inhibition of total culturable anaerobes was observed with 42 μmol/mL NPA and NPOH, with viable cell counts being decreased as much as 90% compared to untreated populations (31). In the case of the barley forages in the present study, only the mixed cultures incubated with the high nitrate-containing barley in experiment 1 had accumulated lower concentrations of total volatile fatty acids than the cultures incubated with alfalfa. These observations suggest that in mixed cultures in experiment 1, having not been adapted to nitrate or inoculated the with nitrate/nitrite metabolizing *D. detoxificans*, the rate of nitrate metabolism to nitrite may have exceeded the rate of nitrite metabolism to ammonia, thus allowing nitrite to temporarily accumulate to levels inhibitory to fiber degrading microbes.

Acetate production by mixed cultures of ruminal microbes is often decreased, and production of more reduced fatty acids, such as propionate and butyrate, are often increased when methane production is inhibited. In both of the present experiments, acetate concentrations in mixed cultures incubated with the nitrate- and nitro-containing forages were lower than in mixed cultures incubated with alfalfa, but this was not always associated with lower methane accumulations. Decreased acetate production in mixed cultures incubated with the NPA- and NPOH-containing forages is not surprising, considering that hydrogen accumulations in these incubations exceeded 1 kPa, which is sufficient to inhibit reoxidation of reduced nucleotides produced during glycolysis (55, 56). Microbial populations often compensate to the accumulation of reduced nucleotides resulting from methane inhibition by redirecting electrons to more reduced acids. Unexpectedly, however, concentrations of propionate were never higher in mixed cultures incubated with the nitrate- and nitro-containing forages than in the mixed cultures incubated with alfalfa. Ratios of acetate to propionate were lower in the mixed cultures incubated with the nitrate- and nitro-containing forages than mixed cultures incubated with alfalfa, due mainly to lower accumulations of acetate. Conversely, butyrate concentrations were almost always higher in the mixed cultures incubated with the nitrate- and nitro-containing forages than those incubated with alfalfa, the exception being mixed cultures inoculated with *D. detoxificans* in experiment 2 that were incubated with the high nitrate-containing barley. Thus, it seems reasonable to suspect that reductant was directed toward butyrate production. These results conflict with earlier results reporting that NPA concentrations as high as 20 μmol/mL had little negative effect on accumulations of acetate, propionate, and butyrate in ruminal populations incubated with or without *D. detoxificans* inoculation (10, 11). Experimental conditions differed between the present and the earlier experiments, however, which confound comparisons with the present experiments.

It is possible reductant was also directed toward the production of valerate in the mixed cultures incubated with the nitrate- and nitro-containing forages of the present experiments. Concentrations of this fatty acid, often associated with protein catabolism, were not higher in the mixed cultures incubated with alfalfa, which is contrary to that expected, due to the alfalfa forage having the higher crude protein content (Table 1). Concentrations of ammonia were higher in the mixed cultures incubated with alfalfa than most of the cultures incubated with the nitrate- and nitro-containing forages, which indicates that protein catabolism was indeed higher in mixed cultures incubated with alfalfa. The main exception being the high accumulations of ammonia observed in the mixed cultures in both experiments 1 and 2 that were incubated with the high nitrate-containing barley, but this is likely due to the near complete reduction of the more than 11 μmol nitrate/mL potentially available in these incubations to ammonia. Accumulations of iso-butyrate and iso-valerate, also associated as potential end products of protein catabolism, were higher in the mixed cultures incubated with alfalfa than in some but not all of the mixed cultures incubated with the nitro- and nitrate-containing forages.

## Conclusion

Forages containing NPA, NPOH, or nitrate effectively decreased methane production during fermentation by mixed cultures of ruminal microbes compared with that produced by mixed cultures incubated with alfalfa, although under the conditions of this experiment these forages caused compensatory changes in fermentation. Inoculation of the mixed cultures with *D. detoxificans*, a ruminal bacterium known to metabolize nitrate, NPA, and NPOH, caused further decreases in methane production during with some but not all of the nitro- or nitrate-containing forages indicating that the *D. detoxificans* effect was not necessarily due to enhanced consumption of reducing substrates. These results will serve as a foundation for continued investigations regarding the inhibitory effects of nitrate and the nitrocompounds on rumen methanogenesis which ultimately may allow formulation of anti-methanogenic diets containing safe amounts of nitrate-, NPA-, or possibly even NPOH-containing forages.

## Author Contributions

Drs. RA, LR, TC, KG, RH, and DN contributed to the design, to the conduct of the experiment as well as to the analysis and interpretation of the results and to writing the manuscript. Dr. RB conducted volatile fatty acid analysis and contributed to interpretation of results and writing the manuscript. Dr. JB contributed the barley forages and to the analysis and interpretation of the results and to writing the manuscript.

## Conflict of Interest Statement

The authors declare that the research was conducted in the absence of any commercial or financial relationships that could be construed as a potential conflict of interest.
